# Is Patient-Provider Racial Concordance Associated with Hispanics’ Satisfaction with Health Care?

**DOI:** 10.3390/ijerph16010031

**Published:** 2018-12-24

**Authors:** Tunay Oguz

**Affiliations:** Charles M. Snipes School of Business and Economics, Lenoir-Rhyne University 625 7th Ave NE, Hickory, NC 28601, USA; tunay.oguz@lr.edu; Tel.: +1-828-328-7807

**Keywords:** health care equity, disparities, policy, patient-provider concordance, acculturation

## Abstract

This study adds a gender approach to determine how patient provider racial concordance and acculturation affect Hispanics’ satisfaction with care and inform more nuanced approaches to improving the quality of care for this population. Using the Medical Expenditure Panel Surveys (MEPS) from 2009–2011, four binary satisfaction outcome measures were created from the MEPS: “doctor showed respect”, “spent enough time”, “explained things in a way you could understand”, and “listened carefully”. Next, a Probit model was employed to estimate the impact of racial concordance and acculturation on the probability of being satisfied with provider care for both male and female Hispanics. For Hispanic women, no significant association was found for the relationship between patient-provider concordance and the overall satisfaction with their care. Hispanic men were found to be less likely to be satisfied with some aspects of their medical care when they were racially concordant with their provider. Overcoming assumptions about shared identity is a crucial step in providing culturally competent care for all patients. There is a need for additional considerations in medical training to help physicians connect with patients, regardless of any type of observable concordance.

## 1. Introduction

The disparities in health care experienced by racial and ethnic groups in the United States are well documented. Numerous studies suggest that minorities are less likely to be insured, less likely to have a regular source of care, and less likely to receive timely and needed care compared to their white counterparts [1,2,3]. Interest in ways to reduce the healthcare disparities faced by racial and ethnic minorities has driven the debate over whether or not creating a more representative healthcare workforce will result in higher satisfaction among minority patients, better access to care, and more positive health outcomes for these groups.

The ways in which increasing the diversity of the healthcare workforce may help improve healthcare access and, subsequently, the health outcomes of minorities have been detailed in a report by the Health Resources and Services Administration (HRSA) [4]. This report posits that minority physicians may possess culturally specific knowledge and experience that may reduce obstacles to patient-physician communication for minority patients. Therefore, increasing the diversity of the healthcare workforce may result in better communication between patients and providers and a higher level of trust in a healthcare system staffed by larger numbers of minority providers [4].

Some studies have found evidence supporting the underlying hypothesis of the HRSA [4] report. These studies have demonstrated that racial and ethnic concordance between patients and providers may lead to a higher likelihood of return for follow up care [5], longer visits [6], and a greater likelihood of using healthcare services [7,8]. Studies have also shown that patient-provider race concordance is positively associated with a greater likelihood of satisfaction with provider care among Black and white patients; however, there is a lack of empirical evidence to support this proposed relationship for Hispanics. Furthermore, the empirical findings for Hispanics and their satisfaction with racially and ethnically concordant providers are mixed. For instance, studies have shown that although Hispanics are more likely than any other ethnic group to rely solely on their provider’s medical advice rather than seeking out information themselves [9], they are also less likely to be satisfied with the overall health care they receive, and in some cases, even racial and ethnic concordance with their providers may not predict Hispanics’ satisfaction [10,11]. 

Mixed findings for Hispanics point to the need for further investigation. Given that policies focused on reducing disparities in health care may be determined based on the assumption that concordance is good for patient-provider relationships, more work is needed to inform resource allocation based on evidence from further empirical research. Using the household component of the 2009, 2010, and 2011 Medical Expenditure Panel Surveys, this study explores the relationship between racial concordance and how Hispanics perceive the quality of their health care (using non-Hispanic whites’ perception of satisfaction for comparison). The goal is to provide greater insight into how patient provider racial concordance contributes to Hispanics’ satisfaction with the health care they receive from their providers, including a consideration of how Hispanic men and women may perceive patient provider racial concordance differently.

## 2. Background

Research studies frequently show that Hispanics are dissatisfied with the medical care they receive from their provider [11,12]. In addition, Hispanics are the largest and fastest growing minority population in the United States. As a result, there has been an increased emphasis in the research literature on the need to better understand the effects of patient provider racial concordance on Hispanics’ satisfaction with medical care. Racial concordance is an important factor impacting the patient-provider relationship and patient satisfaction. Fundamental to the hypothesis supporting racial concordance is the notion that this concordance may result in improved communication. Quality communication is important in clinical settings because it may increase the accuracy of shared information and the quality of care, which could lead to more appropriate diagnoses and treatments which may in turn improve outcomes for minority individuals [13,14].

The impact of patient provider racial concordance on Hispanics’ satisfaction with medical care is often studied from two approaches: examining whether Hispanics report higher levels of satisfaction with care when they are racially concordant with their providers and determining the effect of patient acculturation on patient-provider interactions. 

Regarding the effects of racial concordance, the research findings are inconclusive [15,16]. Some studies have found a significant positive association between patient provider racial concordance and Hispanics’ satisfaction with their medical care [7,17]. For instance, using the 1994 Commonwealth Fund’s Minority Health Survey, LaVeist and Nuru-Jeter [7] and Saha et al. [17] found that being in concordance was associated with a higher level of Hispanic patients’ satisfaction with their provider care compared with patients whose providers who were not racially concordant. Other studies have shown no significant association or weak association of racial concordance on Hispanics’ satisfaction [16,18,19,20]. While Blanchard et al. [18] found that Hispanics in general reported feeling disrespect by racially concordant providers, Schnitter and Liang [16] found that concordance had a positive impact on Hispanics’ perception of racism in the medical encounter, but only for those Hispanics who preferred racially concordant providers. Furthermore, Saha, Arbelaez, and Cooper [19], using various satisfaction measures from the 2001 Commonwealth Fund’s Health Care Quality Survey, found that racial concordance only improved Hispanics’ satisfaction for the amount of time spent with their provider, but racial concordance did not impact Hispanics’ probability of using health services. Finally, Martin, Shi, and Ward [20], using the 2003 Medical Expenditure Panel Survey, found that racial concordance did not predict the rating of the quality of providers’ communication. 

These mixed findings may be due to differences in the ways that studies measured satisfaction. Studies have used patient evaluations of quality of provider care as a measurement of patient satisfaction with the overall care they received, such as whether or not the provider asks questions about other treatments, involves the patient in decision making, shows respect, explains treatment options, spends enough time, treats the patient with dignity, and is accessible by phone or in-person. Not only are the questions changing, how researchers have constructed satisfaction measures varied as well. Some studies have created an index of patients’ rankings of how well their provider performed by compiling questions from the survey they used [7], while others directly asked patients how satisfied they were with the quality of medical care and with their provider [17]. The ways in which researchers have constructed satisfaction measures has led to different conclusions, even when the researchers have used the same data source [7,17]. Mixed findings indicate the complex nature of using the term “satisfaction” to describe a multidimensional phenomenon.

The degree to which acculturation affects patient health behavior is a relatively new area of research, and only a few studies have explored the impact of acculturation on Hispanics’ satisfaction with care [19,21]. For instance, Villani and Mortensen [22] found acculturation to be a significant contributor to the differences in satisfaction between English and Spanish-speaking Hispanics. As with racial concordance, the measurement of acculturation also varies across studies. Some studies use English proficiency as a proxy for Hispanics’ acculturation [15,21]. However, both Cruz et al. [23] and Wallace et al. [24] argue that acculturation cannot be measured using language proficiency alone. Some studies [22,23] have developed acculturation scales that include language preference, such as language used in the interview and language used at home, along with generational status (i.e., first generation or not) and proportion of life lived in the United States. 

This study contributes the literature by adding a gender approach to patient provider racial concordance and acculturation to provide a more nuanced examination of Hispanics’ satisfaction with care. Including a gender approach for Hispanics is necessary to explore how Hispanic men and women differ in their perceptions of the quality of their health care. The hypotheses guiding this study are:

H1: Racial concordance is positively associated with satisfaction with provider care for both Hispanic men and women.
H2: A higher level of acculturation is positively associated with satisfaction with provider care for Hispanic men and women.

## 3. Methods

### 3.1. Data

To test these hypotheses, this study makes use of the household component of the Medical Expenditure Panel Survey (MEPS) to analyze the impact of provider and patient racial and gender concordance on patient satisfaction with provider care. Data used in this study come from combining the household component of the MEPS 2009, 2010, and 2011 (the sampling designs are taken into account in the analysis), limiting the sample to Hispanic and Non-Hispanic white respondents who had a provider visit in the 12 months prior to the survey and those who were between the ages of 18 and 65 at the time of the survey. The sample cutoff age of 65 was chosen because older patients generally have access to Medicare, and they may have more age-associated medical problems requiring more frequent interactions with providers, which could bias the results. The final sample size includes 4,861 non-Hispanic white women, 3,830 non-Hispanic white men, 1611 Hispanic women, and 1212 Hispanic men for a total of 11,514 respondents. 

### 3.2. Outcome Variables

The MEPS asks respondents who had a healthcare visit in the year prior to the survey several questions to assess their satisfaction with their medical care provider. These questions are: “In the last 12 months, how often did doctors or other health providers 1) listen carefully to you, 2) explain things in a way you could understand, 3) show respect for what you had to say, and 4) spend enough time with you?” The possible responses are: “never,” “sometimes,” “usually,” and “always.” Previous studies have frequently converted patient rankings in each category into binary outcome variables (where always = 1 and usually, sometime, and never = 0) to estimate the probability of being satisfied with provider care. This study follows this convention by using binary measures and adopting a Probit model to estimate the probability of being satisfied with provider care.

### 3.3. Explanatory Variables

Aday and Andersen [25] and Andersen’s [26] behavioral model was used to guide the selection of additional explanatory variables that may have an impact on patient satisfaction, including age, marital status, education, English proficiency, perceived physical and mental health status, employment status, income status, health insurance coverage, place of residence, and metropolitan statistical area status (MSA). The study also controlled for fixed effects for survey years to adjust for any possible annual shocks (e.g., policy changes or natural disasters). The MEPS categorizes race and ethnicity as Hispanic (i.e., Puerto Rican, Cuban/Cuban American, Dominican, Mexican/Mexican American, Central or South American) and non-Hispanic white. Respondents also report their provider’s demographic information in such categories as racial background and gender (as perceived by the patient). This allowed for the construction of four binary variables: a racial concordance explanatory variable (1 is racial concordance between the patient and provider, and 0 is otherwise) and a gender concordance explanatory variable (1 is gender concordance between the patient and provider, and 0 is otherwise) for each of the populations under consideration here: non-Hispanic white women, non-Hispanic white men, Hispanic women, and Hispanic men.

Following Villani and Mortensen [22], the language of the interview and the proportion of life a respondent has lived in the United States was used to measure acculturation. If a respondent completed the interview in English only, it was assumed the respondent was comfortable speaking English (as opposed to Spanish or both Spanish and English). The MEPS also asks respondents how long they have been in the United States. Respondents born in the U.S. were assigned a 1, and foreign born were assigned a proportion based on their time spent in the United States divided by their age reported at the time of the survey. 

The MEPS collects comprehensive information on the health insurance coverage of respondents and reports whether or not a respondent was covered by public insurance or private insurance during each month of the year. The MEPS classifies coverage such as Tricare, Medicare, Medicaid or State Children’s Health Insurance Program SCHIP, or other public hospital and physician programs under public insurance. In this study four health insurance categories were created to measure the duration and type of health insurance coverage that each respondent had during the full year: fraction of private coverage, fraction of public coverage, fraction of both private and public coverage, or fraction of uninsured during the full year. For instance, a value of 1 for private coverage indicates that the respondent was fully covered over the full year.

## 4. Econometric Analysis

This study used a Probit model to test whether or not racial concordance and level of acculturation influences patient satisfaction with their provider significantly and by how much. The expected probability can be calculated by using the following equation:

(1) $$Satisfaction_{i}^{*}=S_{i}^{*}=x_{i}^{\prime }\beta +\epsilon_{i}\;\epsilon_{i}\approx N\left(0,\;\sigma_{1}^{2}\right)$$

where $S_{i}^{*}$ represents the unobserved latent variable for individual *i*, and $x_{i}$ is a vector of factors such as individual characteristics, including race (Model 1), age, marital status, region, income, education, insurance status, employment, proportion of life lived in the U.S. and the interview language (Model 2), and patient-provider racial and gender concordance (Model 3). 

The value of the β coefficients determines the relationship between explanatory variables ($x_{i}$) and outcome variables ($S_{i}^{*}$) (i.e β>0 indicates a positive relationship and β < 0 indicates a negative relationship). The $\epsilon_{i}$ is an error term in this equation. $S_{i}=1$ can be observed if and only if $S_{i}>0$ and $S_{i}=0$. The binary choice model is $P\{S_{i}=1\}=F\left(x_{i}^{\prime }\beta \right)$ where F is standard normal distribution. Marginal effects in estimation are reported in results Marginal effects are derived from $f\left(x_{i}^{\prime }\beta \right)\beta$, where $f\left(x_{i}^{\prime }\beta \right)$ denotes the standard normal density function.

## 5. Results

Race and gender summary statistics are provided in Table 1 and Table 2. These statistics show that Hispanics were younger and less likely than non-Hispanic whites to complete the survey in English (up to 65 percent). About 33 percent of Hispanic women and 26 percent of Hispanic men were in the low-income category. Non-Hispanic whites were more educated than Hispanics; up to 25 percent of Hispanics had less than a high school education compared to 7 percent of non-Hispanic whites. The mean duration of private health insurance coverage was about .80 of a 12-month period (equal to 9.6 months) for non-Hispanic whites compared to approximately .60 of a 12-month period (equal to 7.2 months) for Hispanics. All groups reported similar rates of excellent or very good mental health status. However, the tendency to report excellent or very good physical health was lower for Hispanics (80 percent) than for non-Hispanic whites (up to 86 percent).

Table 2 presents satisfaction as measured by responses to the questions listed in the outcome variables section above, demonstrating that Hispanics experience significantly lower satisfaction with their health care than non-Hispanic whites. For instance, Hispanics were less satisfied with the amount of time their healthcare provider spent during a visit compared to non-Hispanic whites. Only about 42 percent of Hispanics reported satisfaction with the amount of time spent with their providers compared to 54 to 56 percent of non-Hispanic whites. Hispanics were relatively less likely to be satisfied with the quality of communication during their visit, as 52 to 54 percent of Hispanics were satisfied with how their healthcare providers explained medical issues to them and listened to them compared to 63 to 66 percent of non-Hispanic whites who reported satisfaction with these aspects. While about 60 percent of Hispanics did report that their healthcare providers always showed respect, this percentage was slightly higher (66 percent) for non-Hispanic whites. Both non-Hispanic white women and Hispanic women reported lower gender concordance (about 37 percent) than non-Hispanic white men (82 percent) or Hispanic men (78 percent). Up to 87 percent of non-Hispanic whites reported that their providers shared the same racial background with them compared to 33 percent of Hispanics. 

Table 3 and Table 4 present the marginal effects for each of the patient satisfaction with care variables. Unadjusted marginal effects are reported in Model 1 (the reference group is Non-Hispanic white men because this population is less likely to experience discrimination [27] and more likely to experience racial concordance with their providers [16,19]. Results from Model 1 suggest that non-Hispanic white women’s satisfaction was not different from that of non-Hispanic white men in general (the only exception is that non-Hispanic white women were less likely to be satisfied with their provider’s listening skills). Hispanic men were consistently less likely than non-Hispanic white men to be satisfied with provider care across each of the patient satisfaction variables considered here. The only exception to this trend was the amount of respect shown by their provider; the probability of Hispanic men being satisfied with this aspect of care was not statistically different from that of white men. Hispanic women were also less likely to be satisfied with the amount of time spent by their providers and their provider’s listening skills compared to non-Hispanic white men. However, these patterns disappeared for both Hispanic men and women in the categories of listening, explanation, and respect when controlling for the other explanatory variables in Model 2.

The results in Model 2 also suggest that older respondents (those in the 50–64 age category) were more likely to be satisfied with their doctor’s listening skills, the amount of respect shown to them by their provider, and the amount of time spent during the examination than their younger counterparts (those in the 18–34 age category). Respondents who had health insurance were more likely to be satisfied with the quality of their medical care compared to those who lacked insurance. When compared to those with low income, having a higher income only improved some aspects of satisfaction with care, specifically, the probability of being satisfied with the explanation of aspects of their medical care and the amount of respect shown to them by their provider. Results support the second hypothesis (H2), in that higher levels of acculturation (measured as proportion of life lived in the United States) was positively associated with satisfaction with care. Acculturation and having excellent or very good mental and physical health increased the probability of being satisfied with the quality of medical care (14 to 19 percentage points and up to 9.7 percentage points respectively).

When controlling for racial and gender concordance in Model 3, the results support the hypothesis (H1) that non-Hispanic whites who have racial concordance with their providers were more satisfied with the quality of their medical care than their non-concordant white counterparts. For instance, with racial concordance, non-Hispanic whites were more likely to be satisfied with the respect shown to them by their provider (6.5 percentage points for women, 7.5 percentage points for men), and they were more likely to be satisfied with the explanation of aspects of their medical care (6.2 and 13.1 percentage points respectively). For the amount of time spent during the examination, both non-Hispanic white women and men were satisfied with their concordant provider (5.5 and 12.0 percentage points respectively), while satisfaction with a doctor’s listening skills was only significant for non-Hispanic white women (7.5 percentage points). However, neither non-Hispanic white men nor non-Hispanic white women reported greater satisfaction (as measured by any of the satisfaction variables) with their care when experiencing gender concordance. 

Findings do not support the hypothesis concerning racial concordance (H1), as results indicate that racial concordance did not have a significant effect on Hispanic women’s satisfaction with provider care. Racial concordance did have a significant effect on satisfaction with care for Hispanic men but in a direction contradicting the first hypothesis. Specifically, when Hispanic men were racially concordant with their providers, they were less likely to be satisfied with their provider’s listening skills (11.8 percentage points) and explanations of aspects of their medical care (10.7 percentage points) than their non-concordant counterparts.

## 6. Discussion

Policy makers and researchers suggest that improving minority representation among physicians in the United States may help reduce barriers to both access to health care and health disparities for minority groups. However, little is known about how achieving such representation (and therefore increasing the chances of concordance) would affect the medical care experiences of minorities. The research findings are mixed on the effects of concordance, and little improvement in patient satisfaction based on concordance has been demonstrated for Hispanics. 

This study hypothesized that Hispanics were more likely to be satisfied with their medical care when they had racially concordant providers. The findings in this study contradict this hypothesis. Although Hispanics in general have been found to prefer racially concordant providers [28,29], this study found that experiencing concordance did not lead to overall satisfaction for Hispanics. 

Hispanic men with racially concordant providers were more likely to be dissatisfied with the listening skills and explanation aspects of their medical care (but not for amount of time spent) than those Hispanic men who were non-concordant. Unlike Blanchard et al. [18], the current study found no association between being in concordance and a higher likelihood of reporting a lack of respect from their provider for Hispanic men (or women). It appears as if Hispanic men’s dissatisfaction with their racially concordant providers is most strongly related to the communication aspects of provider care, so a potential remedy to their reported dissatisfaction needs to address how providers are communicating with members of this population, regardless of concordance. Further research needs to determine what aspects of cultural competency are important to deliver quality of care for diverse populations. Additionally, policies relying on assumptions of concordance based on observable characteristics need to be developed further to include consideration of the heterogeneity of minority populations and account for the complexity of individual identity. A focus on cross-cultural training for medical providers may help to engender a more nuanced and effective approach for providers serving members of minority populations.

The findings also suggest that there is a lack of homogeneity between Hispanic men and women. For Hispanic women there was no significant association found between racial concordance and overall satisfaction with their care for any of the satisfaction measures explored here. Although Hispanic women’s overall satisfaction with their medical care was not found to be associated with racial concordance with their provider, further research into this specific population should examine the ways to improve their satisfaction with their medical care. Differences in results between Hispanic men and women point to the inadequacy of relying on generalizations when studying various populations. Studying minority populations as broad categories overlooks variations within these populations and confounds individual differences; therefore, recommendations need to promote strategies that best meet the needs of individuals rather than those aimed at general population characteristics.

Confirming the second hypothesis of this study, acculturation was found to be positively associated with Hispanic men and women’s satisfaction with their medical care, as those who have spent a greater proportion of their lives in the U.S. reported higher levels of satisfaction with their medical care than less acculturated individuals. An important implication of this finding is that it stresses the need for more culturally competent care for recent immigrants, as lower levels of acculturation had a negative impact on the level of satisfaction with care. These individuals may have expectations for medical care that are still being influenced by conditions in their country of origin. Understanding how this affects the patient/provider relationship is important, because it could lead to the incorporation of this knowledge into models for patient/provider encounters. This implication goes beyond concordance because it emphasizes that the connection between care and cultural competence may be more important than mere concordance between patient and provider.

This study has several limitations. This study is based on cross-sectional data; therefore, inferring causality is not possible. Longitudinal studies are needed to further explore Hispanics’ satisfaction with their racially concordant providers. In addition, the study sample only includes respondents who have had a doctor’s visit in the prior year, which suggest that results may not be applicable to those respondents who didn’t have a doctor’s visit in the same period. Furthermore, since elderly populations (age 65 and above) were not included in the sample of this study, additional research should explore how elderly Hispanics experience medical care with their racially concordant providers. Limitations also include the fact MEPS asks respondents to report their providers’ race, introducing the possibility of misidentification and recall bias. However, the MEPS is more periodic than other national surveys, which perhaps reduces recall bias [30]. Finally, differences by Hispanic ethnic subgroups are not discussed in this analysis. 

## 7. Conclusions

Findings indicate that there are nuances in care preferences that go beyond the idea that patient provider racial concordance will improve the experiences of minorities and eventually contribute to reducing health disparities among minority populations. Working to diversify the healthcare workforce will undoubtedly have benefits. However, doing so is effectively more complicated than previously thought. Overcoming assumptions about shared identity is a crucial step in providing culturally competent care for all patients, and Hispanics are no exception. There is a need for additional considerations in medical training to help physicians identify more strongly with their patients, despite any type of observable concordance. Taking into account the preferences, needs, and values of each individual patient, regardless of their racial background, is necessary to ensure that existing policies targeting ways to improve the quality of health care for minorities are effective.

## Figures and Tables

**Table 1:** Characteristics of Study Sample.

Explanatory Variables	White, Non-Hispanic		Hispanic	
	Women	Men	Women	Men
Sample Size	4861	3830	1611	1212
**Age range**				
18–34	21.2	19.5	32.4	24.5
35–49	30.7	27.8	36.1	39.0
50–65	48.0	52.5	31.4	36.3
**Marital Status**				
Married	62.3	65.5	55.3	58.0
Widowed/Divorced/Separated	19.6	12.6	20.5	14.7
Never married	17.9	21.8	24.2	27.2
**Income Status**				
Low Income	18.7	16.1	33.0	26.8
Middle Income	28.6	25.8	35.7	33.8
Higher Income	52.6	58.0	31.2	39.3
**Education**				
Less than high school	5.2	7.5	21.4	25.2
High school	46.4	44.9	46.8	43.4
More than high school	48.2	47.6	31.6	31.3
Region and MSA* status				
MSA	82.2	83.4	92.9	93.2
West	16.7	15.6	31.8	27.7
Northeast	22.1	23.0	18.0	20.3
Midwest	24.9	25.4	8.7	12.9
South	36.1	35.8	41.3	38.9
**Insurance status**				
Private insurance	0.78	0.80	0.62	0.68
	(0.392)	(0.379)	(0.466)	(0.450)
Public insurance	0.09	0.08	0.18	0.13
	(0.286)	(0.270)	(0.368)	(0.335)
Both private and public insurance	0.03	0.04	0.02	0.03
	(0.163)	(0.186)	(0.141)	(0.159)
Uninsured	0.08	0.07	0.16	0.14
	(0.250)	(0.234)	(0.337)	(0.330)
**Perceived Health Status**				
Excellent or very good physical health	86.8	85.1	80.0	80.0
Excellent or very good mental health	91.2	91.7	90.3	89.8
**Employment**				
Employed	71.6	78.3	68.6	76.5
Unemployed or not in the labor force	28.4	21.6	31.3	23.4

Notes: Authors’ calculations from the Medical Expenditure Panel Survey, 2009, 2010, and 2011. Standard deviations in parentheses. Standard deviations are only reported for continuous measures. Weighted Estimates are reported. * Metropolitan statistical area status (MSA).

**Table 2:** Provider Concordance, Acculturation and Satisfaction Measures by Race and Gender.

Concordance, Acculturation and Satisfaction Measures	White, Non-Hispanic		Hispanic	
	Women	Men	Women	Men
Sample Size	4861	3830	1611	1212
**Doctors or other health providers always**				
showed respect	66.4	65.2	59.8	59.5
spent enough time	54.6	56.7	42.6	42.7
explained things in a way you could understand	63.7	61.6	54.2	52.3
listened carefully to you	63.0	66.0	53.2	54.2
**Concordance and Acculturation**				
Patient-provider gender concordance	37.8	82.0	36.4	78.4
Patient-provider racial concordance	87.2	87.7	31.6	33.2
Proportion of life has lived in the U.S.	0.98	0.98	0.78	0.77
	(0.093)	(0.093)	(0.286)	(0.017)
Interview in English only	99.9	99.7	66.4	64.4

Notes: Author’s calculations from the Medical Expenditure Panel Survey, 2009, 2010, and 2011. Standard deviations in parentheses. Standard deviations are only reported for continuous measures. Weighted Estimates are reported.

**Table 3:** Determinants of Patient Satisfaction with Provider Care.

Determinants of Patient Satisfaction with Provider Care	Doctor Listened			Doctor Explained so Understood		
	Model 1	Model 2	Model 3	Model 1	Model 2	Model 3
Non-Hispanic white women	−0.034 **	−0.034 **	−0.055	−0.002	−0.000	0.069
	(0.014)	(0.014)	(0.043)	(0.012)	(0.012)	(0.043)
Hispanic men	−0.096 **	−0.041	−0.048	−0.073 **	−0.023	0.077
	(0.026)	(0.028)	(0.063)	(0.027)	(0.032)	(0.057)
Hispanic women	−0.059 **	−0.003	0.028	−0.035	0.020	0.139 **
	(0.021)	(0.024)	(0.044)	(0.022)	(0.025)	(0.038)
Racial Concordant white women			0.073 **			0.062 **
			(0.027)			(0.028)
Racial Concordant white men			0.047			0.131 **
			(0.034)			(0.035)
Racial Concordant Hispanic men			−0.118 **			−0.107 **
			(0.044)			(0.042)
Racial Concordant Hispanic women			0.023			-0.008
			(0.032)			(0.039)
Gender Concordant white women			−0.011			−0.025
			(0.020)			(0.021)
Gender Concordant white men			−0.003			−0.000
			(0.026)			(0.028)
Gender Concordant Hispanic men			0.102 *			0.052
			(0.053)			(0.053)
Gender Concordant Hispanic women			−0.015			−0.055
			(0.033)			(0.038)
Proportion of life lived in US		0.158 **	0.150 **		0.180 **	0.172 **
		(0.042)	(0.043)		(0.045)	(0.045)
Interview in English		0.014	0.009		−0.025	−0.032
		(0.033)	(0.034)		(0.034)	(0.035)
Age 35–49		0.015	0.016		0.016	0.019
		(0.019)	(0.019)		(0.022)	(0.022)
Age 50–64		0.043 **	0.044 **		0.032	0.033
		(0.019)	(0.019)		(0.022)	(0.022)
Married		0.010	0.008		0.024	0.020
		(0.022)	(0.022)		(0.019)	(0.019)
Widowed/Divorced/Separated		0.021	0.020		0.038 *	0.035 *
		(0.025)	(0.025)		(0.021)	(0.020)
Middle income		−0.008	−0.009		0.002	0.000
		(0.018)	(0.018)		(0.017)	(0.017)
High income		0.025	0.025		0.043 **	0.042 **
		(0.019)	(0.019)		(0.019)	(0.018)
High school or GED		−0.010	−0.011		−0.009	−0.010
		(0.023)	(0.023)		(0.022)	(0.022)
More than high school		−0.028	−0.031		−0.003	−0.006
		(0.024)	(0.024)		(0.023)	(0.023)
Private insurance		0.053 **	0.053 **		0.053 **	0.054 **
		(0.024)	(0.024)		(0.024)	(0.024)
Public insurance		0.065 **	0.069 **		0.054 *	0.058*
		(0.032)	(0.032)		(0.032)	(0.032)
Both private and public insurance		0.065 *	0.066 *		0.065 *	0.068 *
		(0.035)	(0.036)		(0.037)	(0.037)
Excellent or very good physical health		0.095 **	0.095 **		0.056 **	0.055 **
		(0.019)	(0.019)		(0.022)	(0.022)
Excellent or very good mental health		0.104 **	0.103 **		0.105 **	0.105 **
		(0.025)	(0.025)		(0.024)	(0.024)
Observations	11,294	11,294	11,294	11,310	11,310	11,310

Notes: Standard errors in parentheses. ** Statistically significant at the 0.05 level and 0.01 level. * Statistically significant at the 0.1 level. Adjusted for year fixed effects, metropolitan statistical area status, place of residence, and employment status. The categories “non-Hispanic white men,” “marital status single,” and “age 18–34” are reference categories.

**Table 4:** Determinants of Patient Satisfaction with Provider Care.

Determinants of Patient Satisfaction with Provider Care	Doctor Showed Respect			Doctor Spent Enough Time		
	Model 1	Model 2	Model 3	Model 1	Model 2	Model 3
Non-Hispanic white women	−0.008	−0.007	0.044	−0.023 *	−0.025 *	0.023
	(0.012)	(0.012)	(0.042)	(0.013)	(0.013)	(0.049)
Hispanic men	−0.038	0.018	0.145 **	−0.130 **	−0.074 **	−0.026
	(0.025)	(0.028)	(0.047)	(0.028)	(0.033)	(0.071)
Hispanic women	−0.019	0.041 *	0.138 **	−0.072 **	−0.012	0.068
	(0.022)	(0.025)	(0.036)	(0.021)	(0.024)	(0.046)
Racial Concordant white women			0.065 **			0.055 **
			(0.029)			(0.027)
Racial Concordant white men			0.075 **			0.120 **
			(0.032)			(0.034)
Racial Concordant Hispanic men			−0.047			−0.057
			(0.040)			(0.048)
Racial Concordant Hispanic women			−0.005			0.029
			(0.040)			(0.038)
Gender Concordant white women			−0.018			−0.023
			(0.020)			(0.020)
Gender Concordant white men			0.043 *			−0.022
			(0.025)			(0.027)
Gender Concordant Hispanic men			−0.041			0.072
			(0.051)			(0.062)
Gender Concordant Hispanic women			−0.031			−0.009
			(0.038)			(0.033)
Proportion of life lived in U.S.		0.147 **	0.142 **		0.194 **	0.192 **
		(0.042)	(0.043)		(0.047)	(0.048)
Interview in English		−0.012	−0.016		−0.010	−0.009
		(0.034)	(0.033)		(0.037)	(0.038)
Age 35–49		0.016	0.019		0.017	0.018
		(0.021)	(0.021)		(0.020)	(0.020)
Age 50–64		0.041 **	0.042 **		0.047 **	0.048 **
		(0.020)	(0.021)		(0.020)	(0.021)
Married		0.008	0.005		0.041 *	0.038 *
		(0.020)	(0.020)		(0.021)	(0.021)
Widowed/Divorced/Separated		0.014	0.011		0.056 **	0.054 **
		(0.023)	(0.022)		(0.022)	(0.022)
Middle income		0.003	0.001		−0.011	−0.012
		(0.017)	(0.017)		(0.020)	(0.020)
High income		0.043 **	0.042 **		0.026	0.025
		(0.017)	(0.017)		(0.021)	(0.022)
High school or GED		−0.011	−0.011		−0.023	−0.023
		(0.024)	(0.024)		(0.026)	(0.026)
More than high school		0.006	0.004		−0.028	−0.030
		(0.023)	(0.023)		(0.027)	(0.027)
Private insurance		0.070 **	0.071 **		0.070 **	0.072 **
		(0.022)	(0.022)		(0.026)	(0.026)
Public insurance		0.076 **	0.079 **		0.055 *	0.058 *
		(0.029)	(0.029)		(0.031)	(0.031)
Both private and public insurance		0.078 **	0.080 **		0.083 **	0.087 **
		(0.039)	(0.039)		(0.042)	(0.042)
Excellent or very good physical health		0.068 **	0.068 **		0.097 **	0.095 **
		(0.021)	(0.021)		(0.023)	(0.022)
Excellent or very good mental health		0.114 **	0.113 **		0.083 **	0.083 **
		(0.024)	(0.024)		(0.025)	(0.025)
Observations	11,319	11,319	11,319	11,270	11,270	11,270

Notes: Standard errors in parentheses. **Statistically significant at the 0.05 level and 0.01 level. *Statistically significant at the 0.1 level. Adjusted for year fixed effects, metropolitan statistical area status, place of residence, and employment status. The categories “non-Hispanic white men,” “marital status single,” and “age 18–34” are reference categories.
